# Revisiting the Neuropathology of Sudden Infant Death Syndrome (SIDS)

**DOI:** 10.3389/fneur.2020.594550

**Published:** 2020-12-17

**Authors:** Jessica Blackburn, Valeria F. Chapur, Julie A. Stephens, Jing Zhao, Anne Shepler, Christopher R. Pierson, José Javier Otero

**Affiliations:** ^1^Division of Neuropathology, Department of Pathology, The Ohio State University College of Medicine, Columbus, OH, United States; ^2^Division of Anatomy, Department of Biomedical Education & Anatomy, The Ohio State University College of Medicine, Columbus, OH, United States; ^3^Instituto de Ecoregiones Andinas (INECOA)/Consejo Nacional de Investigaciones Científicas y Técnicas (CONICET), Buenos Aires, Argentina; ^4^Instituto de Biología de la Altura (INBIAL)/Universidad Nacional de Jujuy (UNJU), San Salvador de Jujuy, Argentina; ^5^Department of Biomedical Informatics, Center for Biostatistics, The Ohio State University College of Medicine, Columbus, OH, United States; ^6^Franklin County Forensic Science Center, Columbus, OH, United States; ^7^Department of Pathology and Laboratory Medicine, Nationwide Children's Hospital, Columbus, OH, United States

**Keywords:** sudden infant death syndrome (SIDS), neuropathology, cluster analysis, infant mortality rate, infant death

## Abstract

**Background:** Sudden infant death syndrome (SIDS) is one of the leading causes of infant mortality in the United States (US). The extent to which SIDS manifests with an underlying neuropathological mechanism is highly controversial. SIDS correlates with markers of poor prenatal and postnatal care, generally rooted in the lack of access and quality of healthcare endemic to select racial and ethnic groups, and thus can be viewed in the context of health disparities. However, some evidence suggests that at least a subset of SIDS cases may result from a neuropathological mechanism. To explain these issues, a triple-risk hypothesis has been proposed, whereby an underlying biological abnormality in an infant facing an extrinsic risk during a critical developmental period SIDS is hypothesized to occur. Each SIDS decedent is thus thought to have a unique combination of these risk factors leading to their death. This article reviews the neuropathological literature of SIDS and uses machine learning tools to identify distinct subtypes of SIDS decedents based on epidemiological data.

**Methods:** We analyzed US Period Linked Birth/Infant Mortality Files from 1990 to 2017 (excluding 1992–1994). Using t-SNE, an unsupervised machine learning dimensionality reduction algorithm, we identified clusters of SIDS decedents. Following identification of these groups, we identified changes in the rates of SIDS at the state level and across three countries.

**Results:** Through t-SNE and distance based statistical analysis, we identified three groups of SIDS decedents, each with a unique peak age of death. Within the US, SIDS is geographically heterogeneous. Following this, we found low birth weight and normal birth weight SIDS rates have not been equally impacted by implementation of clinical guidelines. We show that across countries with different levels of cultural heterogeneity, reduction in SIDS rates has also been distinct between decedents with low vs. normal birth weight.

**Conclusions:** Different epidemiological and extrinsic risk factors exist based on the three unique SIDS groups we identified with t-SNE and distance based statistical measurements. Clinical guidelines have not equally impacted the groups, and normal birth weight infants comprise more of the cases of SIDS even though low birth weight infants have a higher SIDS rate.

## Introduction

Few topics in forensics enwrap themselves in as much controversy as the neuropathology associated with sudden infant death syndrome (SIDS). The interaction of concurrent epidemiological risks often associated with impoverished communities adds a level of complexity to SIDS pathogenesis (1, 2). As SIDS correlates with other markers of poor prenatal and postnatal care, which are generally rooted in the lack of access and low quality of healthcare endemic to impoverished racial and ethnic groups, some have come to view SIDS as a disease of health disparities (3–5). Although these epidemiological associations are undeniable, there is also a compelling case, at least in a subset of SIDS decedents, for a primarily neurological, and potentially neuroanatomical, etiology to the patient's death (6). This has significant impact on health policies since they would require implementation of different solutions to prevent these tragedies. For instance, can SIDS be prevented by abrogating health disparities across society, or should investment in basic, mechanistic research into the biological basis of SIDS be prioritized so that we may identify medical interventions? We posit that these policies would not represent mutually exclusive mechanisms to SIDS etiology. Attempts to determine SIDS events due to neurological mechanisms will emerge as a major research need within forensic neuropathology and may complement existing measures in prevention that have been successful in reducing the incidence of SIDS (7, 8).

SIDS neuropathological research has primarily focused on evaluation of the brainstem, due to its critical role in the regulation of the autonomic nervous system. In 1976, Dr. Neaye identified reactive astrogliosis in the medullary reticular formation in association with SIDS (9). Reactive gliosis often represents a general (often chronic) response to a brain injury and, depending on the context, induces protective or detrimental pathways to modulate neurological function. Although astrocytic gene expression changes occur within 1 hour post-brain injury, morphological changes of astrogliosis manifest along more sub-acute time frames (10). In rodents, middle cerebral artery occlusion induces morphological changes in astrocytes first detectable 2 days post infarction with additional dynamic morphological changes for 14 days (11). Thus, morphologically detectable brainstem astrogliosis in a SIDS decedent may imply a subacute/chronic developmental neuropathological change in an otherwise healthy infant, rather than an acute event such as an accidental asphyxiation. However, it is yet to be determined whether brainstem reactive astrogliosis indicates a primary abnormal developmental neuropathological event or is secondary to hypoxia-ischemia (9, 12, 13). With identification of brainstem gliosis, researchers have extensively focused on brainstem nuclei and their projections to other brain regions that have critical roles in autonomic regulation.

The brainstem serotonergic (5-HT) system regulates respiration and airway patency, homeostatic functions, sleep and arousal (12). The medullary 5-HT system is divided into rostral and caudal domains with projections distributed widely throughout the central nervous system. The rostral 5-HT domain projects to telencephalic and diencephalic structures responsible for cognition and arousal. In contrast, the caudal 5-HT domain projects primarily to effector nuclei in the rhombencephalon such as the nucleus of the solitary tract, pre-Bötzinger complex, phrenic nucleus, hypoglossal nucleus, dorsal motor nucleus of the vagus, and nucleus ambiguus. These nuclei integrate diverse, yet interrelated functions including thermoregulation, cardiovascular homeostasis, inspiratory rhythmogenesis, respiratory rhythm generation, diaphragmatic innervation, and airway patency during sleep. It is hypothesized that these disruptions in the brainstem 5-HT system are due to a combination of genetic and environmental factors. In the event that an infant with an inherent underlying vulnerability during this critical period faces an exogenous stressor, the convergence of faulty reflexes controlled by the 5-HT (and other) systems leads to death. This hypothesis is supported by data showing medullary 5-HT abnormalities in four distinct data sets over a fourteen year period (14–17). Serotonergic abnormalities are shown in 4 additional datasets (18–21), however, as Paine et al. highlighted in 2014, these as well as many neuropathological studies of SIDS use inconsistent definitions or do not use age- and sex-matched controls which vastly limits comparison (13).

Although the 5-HT system is incredibly diverse and spans the central nervous system, the medullary portion is predominantly composed of *Pet1-*expressing neurons which regulate breathing in neonates (22). These neurons regulate the apnea-induced autoresuscitation reflex and the laryngeal chemoreflex in response to obstructive fluids in the airway, both when impaired are hypothesized contributors of SIDS. In addition, in genetic murine models, these neurons are developing in the equivalent adjusted inter-species time frame as the critical period in which SIDS is most likely to occur in humans (2–4 months of age in *Homo sapiens*, postnatal day 1–10 in *Mus musculus*) (22, 23). In summary, there is compelling evidence derived from descriptive human-based tissue research as well as mechanistic data in experimental animals supporting the notion that a primary developmental neurological driver may be implicated in SIDS.

Nevertheless, there are also significant indications of a health disparities component to SIDS etiology. Thirty years ago the rate of SIDS in the United States (US) was considered moderate compared to other developed countries, however the US now has among the highest SIDS and postnatal mortality rates in recent years (24). SIDS, accidental suffocation/strangulation, and other ill-defined or unspecified causes of mortality are three causes of death that fall under the definition of sudden unexpected infant death (SUID). Substantial disparities are evident when comparing the rate of SIDS and SUID amongst distinct racial and ethnic groups in the US (4, 25). For instance, in 2017, 35.4 deaths per 100,000 live births occurred in the US attributed to SIDS^1^. In Native American and American Indian populations, the SIDS rate from 2014 to 2017, was 2.5 times higher than the rate of non-Hispanic white infants (95.59 and 37.65 per 100,000 live births, respectively)^1^. In addition, non-Hispanic Black infants have almost 2 times the rate of SIDS compared to non-Hispanic white infants (73.6 and 37.65 per 100,000 live births, respectively). The extent to which these distinct rates result from intrinsic genetic polymorphisms vs. other social or geographical determinants of health is highly debated in the neuropathology literature (6). It is well-known that discrete epidemiological risk factors exist for these different racial groups in regards to modifiable and non-modifiable behaviors, such as maternal smoking, breastfeeding, prematurity, and infant sleep environment (26, 27). For instance, the Aberdeen Area Infant Mortality Study, which enrolled patients for prospective observation from 1992 to 1996 revealed the following risks (in order of importance): 1st trimester maternal binge drinking, periconceptual maternal drinking, and over-bundling the infant (28). In addition to these risk factors, these decedents were also found to have abnormalities in the 5-HT system (20).

Due to these divergent perspectives, some investigators have advocated for a Triple Risk Hypothesis for SIDS. The Triple Risk Hypothesis posits that a vulnerable infant with an underlying intrinsic risk undergoes exposure to an unsurmountable exogenous stressor resulting in death (29). These intrinsic risks include genetic components such as male sex or potential polymorphisms in the serotonergic pathway, developmental risks such as prematurity, and environmental risks such as perinatal and postnatal exposure to smoking, ethanol, or other drugs (30). Extrinsic risks are physical stressors such as bed sharing, soft bedding, sleeping in non-supine positions, over-bundling, and all other risks that may lead to asphyxia or compromise homeostatic regulation. In summary, each decedent's death is due to an interaction of stressors originating in each of the “Risk Spheres” of this model. A corollary of the Triple Risk Hypothesis is that each death may have different contributions from each risk sphere, such that in some infants death follows mainly intrinsic etiologies, whereas in other infants death would result from extrinsic factors, and so forth.

Although the Triple Risk Hypothesis has represented a useful framework to understand SIDS, the inherent variabilities and confounding factors in studying human tissue make it impossible to predict which individual infant is at clear risk of sudden death. This challenge prevents delineation of clear mechanistic pathways that could be amenable to pharmaceutical intervention. Amongst these hindrances includes an inability to objectively determine the underlying etiology and relative contribution of various extrinsic and intrinsic risks of each individual decedent. Therefore, we sought to identify unique clusters of SIDS decedents in whom different risk factors and characteristics predominate. To achieve this, we obtained data from the United States National Center of Health Statistics (NCHS) managed by the Centers for Disease Control and Prevention and applied modern unsupervised machine learning models.

Neuropathology has evolved into a “big data” field with routine incorporation of machine learning and artificial intelligence informatics that spans a much larger scope than interpretation of histopathological slides. We performed an unsupervised machine learning dimensionality reduction technique known as t-distributed stochastic neighbor embedding (t-SNE). Due to the data recording methods obtained from the NCHS infant mortality files, this method was best suited for our analysis. t-SNE is a high-dimensionality reduction technique capable of modeling non-numerical data in the form of factors (31). Our analysis was narrowed to 16 categorical variables in the t-SNE computation, which permitted us to identify unique clusters of patients which were confirmed by distance statistics. The identification of patients within a heterogenous disease was initially implemented in diagnostic neuropathology in the early 2000s (32–34). This approach has been instructive in neuropathology identifying unique patient subtypes and we utilized similar methods to identify unique clusters of SIDS decedents. By identifying discrete clusters within the t-SNE analysis, we further investigated differences between these groups in terms of post-conceptional age at death and how they have been influenced over time. Lastly, our analysis sought to analyze geographic trends over time between regions and the rates of SIDS between two birth weight groups, and to compare outcomes in the US to other countries with similar or less cultural heterogeneity.

## Materials and Methods

### Data Acquisition and Standardization

Analyses were primarily conducted using the US 1990–1991 and 1995–2017 Period Linked Birth/Death Data Sets by the National Center for Health Statistics (data files were not produced during 1992–1994)^2^. To identify SIDS decedents, we used the *International Classification of Diseases, Ninth Revision* (ICD-9) code for deaths occurring before 1999 (code 798.0) and *International Classification of Diseases, Tenth Revision* (ICD-10) for deaths occurring in 1999 and after (code R95) (35). These data were used to create t-SNE plots and calculate the rates of SIDS based on different variables.

In addition to analyzing US SIDS rates, our study also included calculating the SIDS mortality rates in New Zealand and Argentina. Publicly available New Zealand SIDS mortality data was obtained from the Ministry of Health from 2000 to 2016, and República de Argentina data was obtained from Dirección de Estadísticas e información en salud del ministerio de salud de la nación (Argentine Government) for 1997–2015. The US and Argentina defined SIDS occurring from birth to 1 year of age and New Zealand classified it from birth to <1 year of age in their reporting statistics (24). All countries utilized the same ICD-10 coding standard. Utilizing this metric as an international standard of comparison in SIDS rate has been previously presented by Hauck and Tanabe (24). Argentina, New Zealand and the US yields similar distribution of R95 diagnoses allowing us to trust the validity of this measure (36, 37).

SIDS mortality rates by birth weight were calculated using the total number of SIDS decedents in that birth weight class divided by the number of live infants born in the same birth weight class multiplied by 1,000. SIDS decedents with unknown birth weights were excluded from the rate calculation in analysis of all countries (0.088% excluded from US data, 4.4% excluded from New Zealand Data, 52.5% excluded from República de Argentina data).

For the US data, individual state death rate calculations were derived by dividing SIDS occurrences in a birth weight category by the number of live born infants. To avoid disclosing identifiable events, rates underwent center-scaling at zero with a standard deviation of 1 in each birth weight category. Restricted US Period Linked Birth/Death Data from 2005 to 2006 and 2014–2017 omitted state of death occurrence and were excluded from our analysis.

In order to calculate the adjusted age at death, we used the gestational age recorded in the birth certificate computed using the date of birth of the infant and last menstrual period or the clinical estimate of gestation. We considered 39 weeks gestation or higher as full term as recommended by Spong et al. (38). Gestational age (in weeks) was subtracted from 39, multiplied by 7, and subtracted from the age at death (in days) recorded on the death certificate (*n* = 29,651; 48.51%). Infants with 39 weeks gestational age or higher did not have their age at death modified (*n* = 30,882; 50.53%), nor did the infants who had “unknown” or “not recorded” listed as the gestational age on their birth certificate (*n* = 585, 0.96%).

Mother's education was also recoded due to variations in the reporting codes between the unrevised and revised birth certificates. See Supplementary Table 1 for recoding parameters.

### T-SNE Modeling and Cluster Validation

Traditional methods of studying the epidemiology of SIDS typically focus on the relationship of the event and possible risk factors associated with SIDS and each other (4, 27). To evaluate all variables, we used t-SNE, a high dimensionality reduction technique to visually observe non-linear trends in the infants dying of SIDS (39). Data are put into a model and an individual circle represents a single decedent of SIDS in the US from 1990 to 2017 (excluding 1992–1994). Variables included in our model are listed in Supplementary Table 2. t-SNE plots were pseudo-colored for each variable to identify clustering trends. The x and y axis represent the first and second dimension of the t-SNE analysis and similar data points are plotted near one another. Additional parameter t-SNE plots with varying perplexity and maximum iterations are included in Supplementary Figure 1.

In addition, we performed internal clustering validation to compute the distance between objects and their respective clusters. We started by creating a dummy variable matrix for every column to separate our factors (40). This resulted in a large dataframe (dimensions 61,118 × 128) which was challenging to interrogate with the dist() function in R. We therefore performed a permutation analysis to implement the cluster.stats() function call in R and evaluated the average distance between clusters, the average distance within clusters, and the within cluster sum of squares using the cluster.stats() function call in R. Supplementary Figure 2 discusses our work-flow for this analysis. This permutation was repeated 1,000 times, which permits identifying significance scores of up to 10^−2^ (represented as 10^−2^ due to being an estimate obtained using random sampled permutations) (41).

## Results

### Epidemiological Data From SIDS Decedents Cluster Into Three Groups

**Figure 1:**
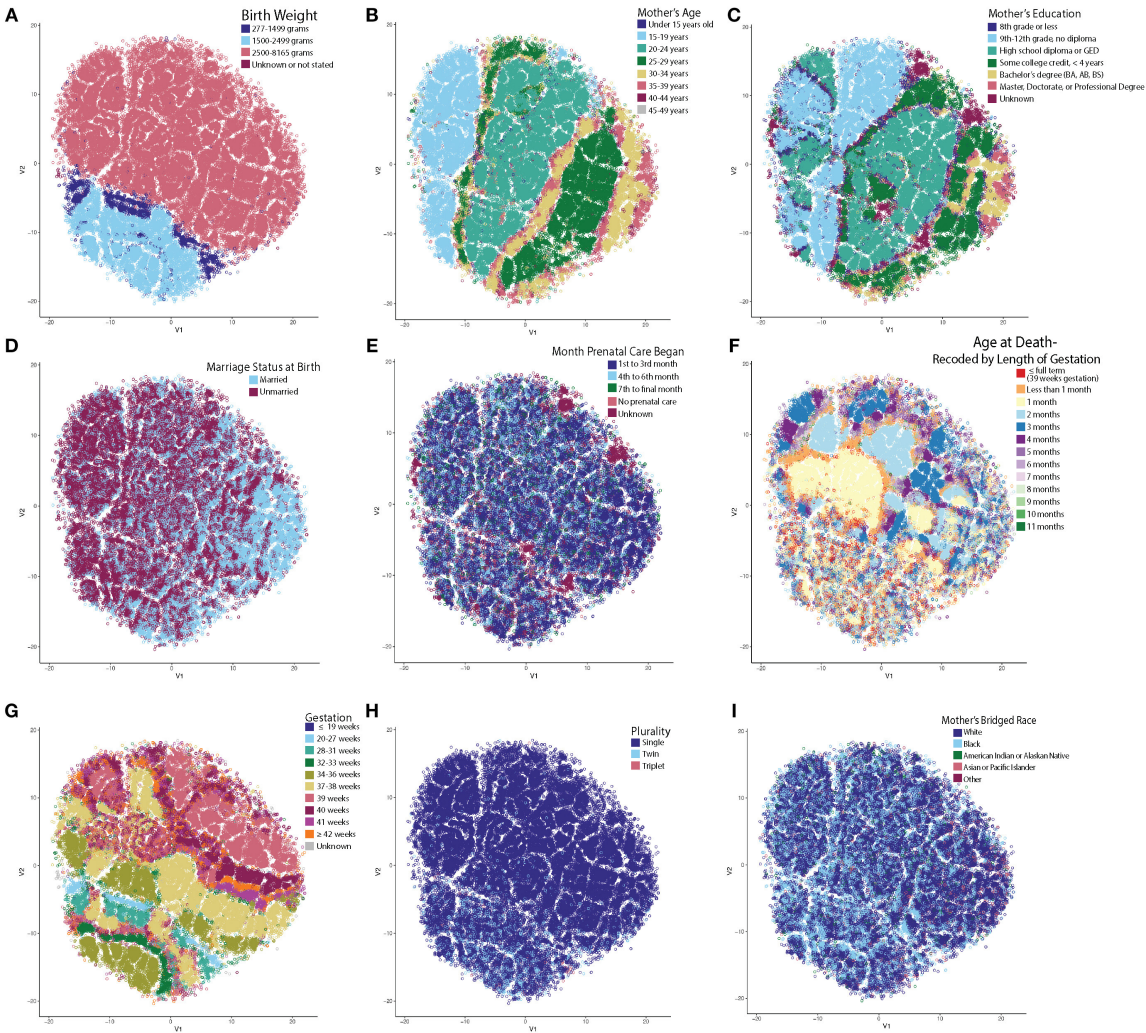
t-SNE Reveals Unsupervised Clustering Patterns. Each circle represents one SIDS decedent and has been pseudo-colored for each variable to identify clustering trends. X and Y axes represent the first and second dimension of the t-SNE analysis and units are arbitrary. **(A)** Infant's birth weight created the most distinct separation of clusters. Very low and low birth weight infants clustered together with very little intermingling between the normal birth weight infants. **(B)** Mother's age at time of birth. **(C)** Mother's highest attained education at time of birth. Note that on the left in B and C, are teenage mothers still in high school compared to the right side of the plot with mothers who are older and have a higher education. **(D)** Mother's marital status at birth. Note that the older moms (30+) are more frequently married. **(E)** Month prenatal care was initiated. **(F)** Age at death recoded by length of gestation. Note that the low birth weight infants and infants who are born to older, more educated, and married mothers have more heterogenous clustering patterns. **(G)** Low birth weight infants dying from SIDS have shorter lengths of gestation and less frequently reach full term gestation (39+ weeks). **(H)** Multiparous pregnancy. Infants in the low birth weight cluster have a higher percentage of twin and triplet occurrences compared to single births. **(I)** Mother's bridged race. It is known that there are large racial disparities in the rate of SIDS. Note the high density of Black mothers in the low birth weight cluster and how there is a larger percentage of mothers who are white in the cluster forming on the right half (identified as group 2 in Figure 2).

By far, the most extensive dataset available for the study of SIDS in the US represents the vital statistics available through the NCHS. We therefore obtained and standardized the Period Linked Birth/Infant Death data to allow for robust analysis of SIDS decedents. These data represented 61,118 instances of SIDS decedents linked to information on both the birth certificate and death certificate, including mother's marital status, education, race, and prenatal care in addition to the infant's length of gestation, birth weight, and age at death. These parameters were chosen because variables including father's education, age, and race were inconsistently reported across the various states, alcohol usage was not collected over the entire timeframe studied, and infants with multiple variables as “unknown” clustered near one another. Clusters based on unreliable reporting led us to exclude these variables. We first tested the hypothesis that unique clusters of SIDS decedents existed by performing t-SNE analysis. Following the creation of the t-SNE plot, we pseudo-colored each circle (where each circle represents one instance of a SIDS decedent). By interacting with the data in this way, we identified trends in the data as to which variable created the most segmented groups. Birth weight was the most significant driver separating the groups (Figure 1A). Infants with very low birth weight (indigo) and low birth weight (light blue) clustered closer together compared to normal birth weight infants (pink), with very little intermingling between groups at the border (Figure 1A). In summary, even when using multiple parameters in the t-SNE function call, birth weight was the most significant driver in group separation (Supplementary Figure 1).

Upon further data exploration, we identified a smaller, second group within the normal birth weight cluster (Figures 1B–E). Although not as easily segregated visually in the t-SNE as the birth weight clusters, we noticed that the t-SNE algorithm clustered decedents whose mothers were 25 years of age or older (Figure 1B), attended college for at least 4 years (or completed a bachelor's degree or higher) (Figure 1C), were married (Figure 1D), and began prenatal care in the first trimester (Figure 1E). Based on known demographic trends, these decedents likely came from families that had more stable financial and home environments. We also noted that a third group, located in the center of the t-SNE plot, showed significant clustering by the adjusted age at death (Figure 1F).

In these three discrete groups, we noticed age at death (Figure 1F) did not show significant clustering within the low birth weight group (group 1) or in group 2 comprised of older, more educated, and married mothers who started prenatal care in the first trimester. In addition, the infants in group 1 had a different pattern of clustering by gestation (Figure 1G) and a larger percentage of infants in this group were from multiparous gestations (Figure 1H). Based on a chi-square test, there was a statistically significant higher percent of white mothers in group 2 than groups 1 and 3 (Figure 1I). We further evaluated the distance between clusters (Supplementary Figure 2), and found that the mean within cluster sum of squares of these three clusters was 45,231 (*SD* = 82.9, *p* < 10^−2^), the mean average within cluster distance was 4.23 (*SD* = 0.0039, *p* < 10^−2^), and the mean average between cluster distance was 4.48 (*SD* = 0.0046, *p* < 10^−2^). We conclude that three discrete groups of decedents were identified by machine learning-based evaluation of the NCHS epidemiological data.

**Figure 2:**
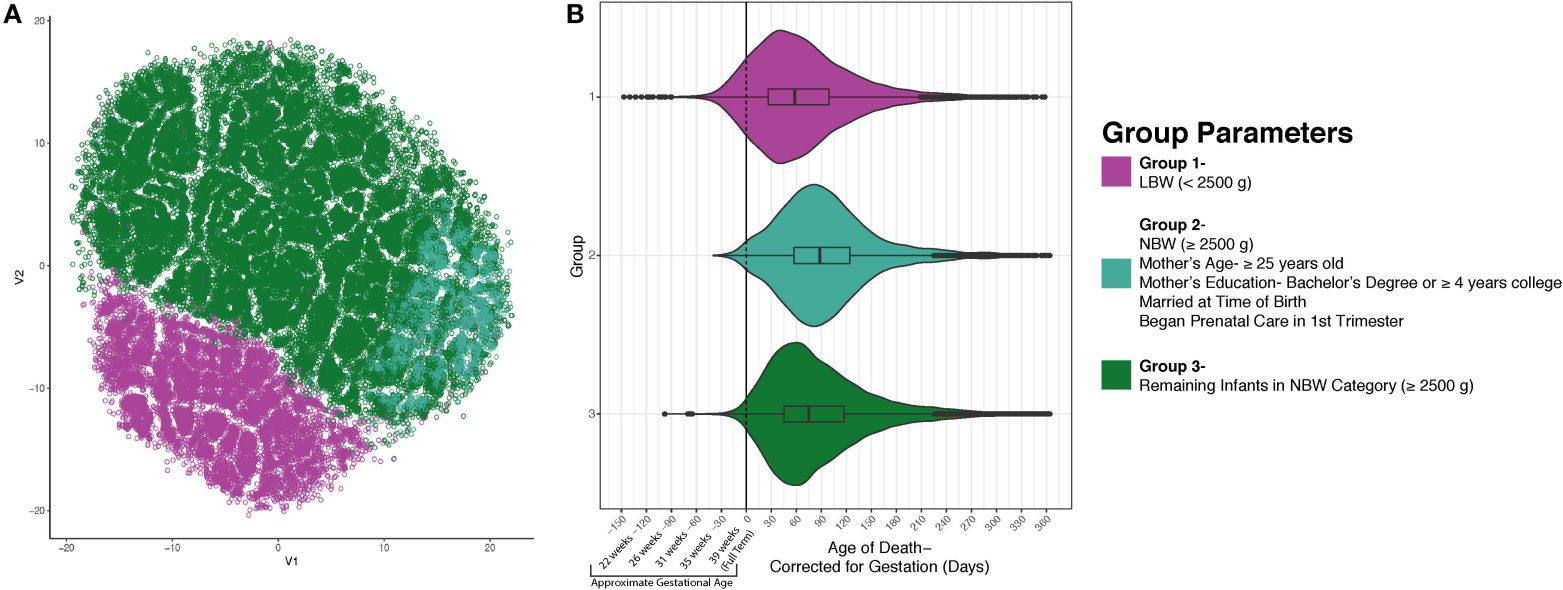
SIDS Decedents Cluster into Three Distinct Groups. After using unsupervised modeling with t-SNE, we identified 3 discrete groups based on infant birth and death data as well as maternal characteristics **(A)**. Group 1 (magenta) is formed by the infants who weighed <2,500 grams at birth. Group 2 is less segregated and found plotted on the right side in teal. This group is comprised of decedents who are normal birth weight and had mothers who are 25 years of age or older, had a bachelor's degree or ≥4 years of college, married, and began prenatal care in the first trimester. Group 3 (green) are the remaining infants in the normal birth weight category. **(B)** After identifying three discrete groups, we sought to identify differences in postconceptional age at death. There is a statistically significant distinction in the average age at death for each group (*p* < 2e^−16^). Nearly 10% of the infants in group 1 passed away before reaching full term gestation which could indicate a different etiology of SIDS.

Having identified these three groups using an unsupervised learning approach, we next sought to identify potential differences between these groups that might provide insight into the etiology of SIDS. In Figure 2A, group 1 (low birth weight) is illustrated in magenta (*n* = 12,310), group 2 (normal birth weight decedents with mothers attaining higher education, 25 years or older, married, and in whom prenatal care commenced in the first trimester) in teal (*n* = 5,143), and group three (remainder of decedents in the normal birth weight category) in green (*n* = 43,665). Following the identification of these three groups, we next tested if the mean age of death was distinct between groups. When comparing the three groups' postnatal age at death based on the death certificate, we noticed that each group had similar peak ages at death. However, since low birth weight is highly related to prematurity, we recalculated each decedent's postconceptional age so that we could plot all decedents along the same timeline. When plotting the three distinct groups based on their postnatal age corrected for gestation, we discovered differences in the distribution and arithmetic mean of age at death between the three groups. In group 1, the mean age at death was 67.89 days (*SD* = 61.95); group 2's mean age at death was 96.80 days (SD = 60.71); and group 3's mean age at death was 88.22 days (*SD* = 62.82). Evaluation of statistical significance by ANOVA/TukeyHSD test demonstrated a significance of *p* < 2e^−16^ and an adjusted *p*-value of 2e^−16^, 2e^−16^, and 3e^−14^, for groups 2-1, groups 3-1, and groups 3-2, respectively. These data indicate that the mean peak incidence of SIDS within these three groups occurs at distinct neurodevelopmental ages. In addition to the groups having significantly different postconceptional ages at death, it is important to note that many infants (~10%) born prematurely in group 1 died of SIDS before they reached full-term gestation with a calculated *p*-value <2e^−16^ using a chi-square test. This larger percentage of infants dying before reaching full term gestation in the low birth weight group could suggest that in this group of SIDS decedents, neurodevelopmental pathologies may play more important roles in SIDS pathogenesis relative to the other groups.

### Geographical Discrepancies of SIDS Mirrors Drivers of Health Disparity in the US

**Figure 3:**
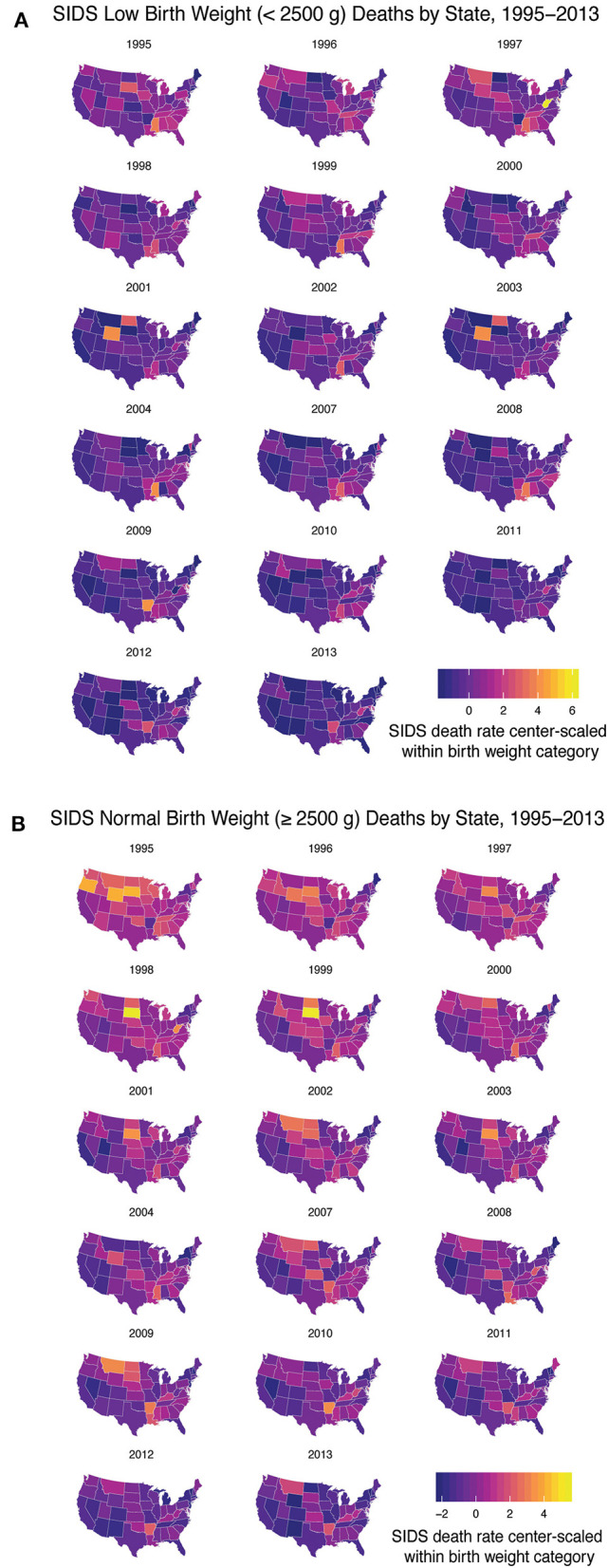
SIDS Rates Disparities Persist Across the US. From 1995 to 2013 (excluding 2005–2006) we analyzed the geographical heterogeneity of SIDS rates based on decedent birth weight. **(A)** Low birth weight infants had a higher rate of SIDS in West Virginia, Arkansas, Wyoming, Mississippi, North and South Dakota, Montana, and Louisiana and the District of Columbia. **(B)** Normal birth weight decedents had higher rates of SIDS mirroring the states that had high rates of low birth weight SIDS. Geographical disparities persisted over time based on infant birth weight.

After determining there were three discrete groups based on maternal and infantile characteristics, we wanted to see how each state performed over time in regards to reducing the rate of SIDS in these groups. Due to limited data availability, we were only able to perform this analysis with group 1 (infants weighing <2,500 g) and a second group based on infants born weighing 2,500 g or greater. Data were center-scaled within birth weight categories including geographical information on SIDS death from 1995 to 2013 (excluding 2005–2006). We found over time, states have generally reduced the rate of SIDS for these two groups, however, disparities are still present. In the low birth weight group, states that had higher rates of SIDS include West Virginia, Arkansas, Wyoming, Mississippi, North and South Dakota, Montana, Louisiana, and the District of Columbia (Figure 3A). In the normal birth weight group, states that had higher rates of SIDS mirrored many of the low birth weight SIDS rates and include South and North Dakota, Wyoming, Oregon, Arkansas, West Virginia, Montana, Mississippi, and Louisiana (Figure 3B). In general, the rates were highest in the Southeast and Northwest regions and lower in the Pacific and Northern Atlantic coastal regions of the US. We also note that high correlation between low birth weight SIDS rate and normal birth weight SIDS rate by state geography (*p* < 0.001), indicating that a strong geographical heterogeneity in SIDS incidence across the US. We also note that regions of SIDS hotspots mirror the geographic distributions of African American and Native American populations as well as regions with lowest GDP per capita. This correlation was determined in two separate linear regression models analyzing the 2013 SIDS rate based on race (i.e., response variable) in each state compared to the GDP (i.e., predictor variable). State GDP shows a significant linear relationship with the rate of white infants dying of SIDS but no correlation with Black infants. The linear regression data are reported in Supplementary Table 3.

### Implementation of Clinical Guidelines Shows Distinct Impact in Decreasing SIDS Rates in Different SIDS Clusters

**Figure 4:**
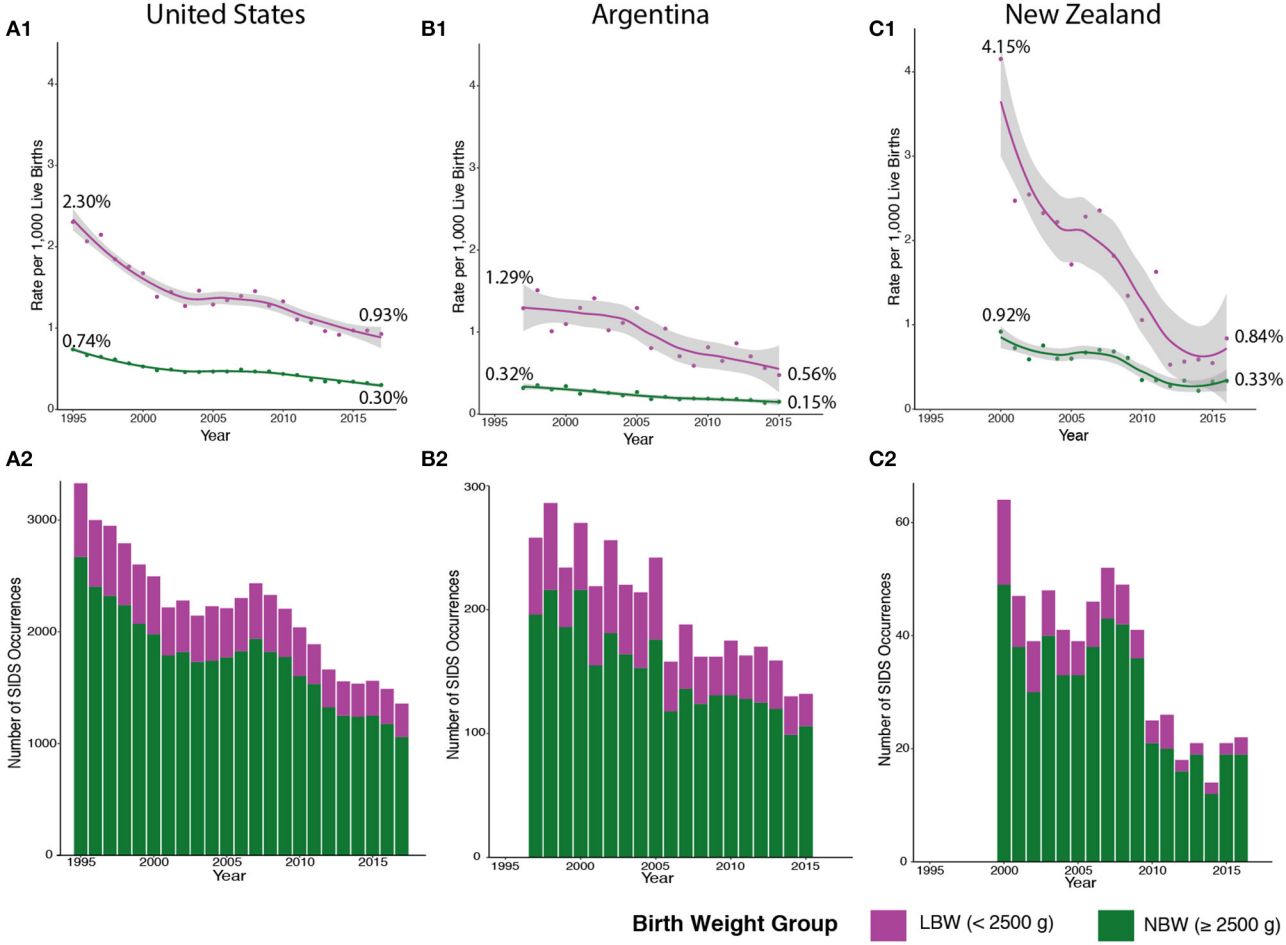
SIDS Rates in the US, Argentina, and New Zealand Follow Similar Trends. We broadened our analysis to include countries with different levels of cultural heterogeneity compared to the US. SIDS rates in low birth weight (LBW) infants (colored in magenta) are higher in the US **(A1)**, Argentina **(B1)**, and New Zealand **(C1)** compared to normal birth weight (NBW) infants (colored in green). There is also a more drastic decline in LBW SIDS rates compared to a stable SIDS rate in NBW infants. However, fewer cases of SIDS are comprised of LBW infants in the US **(A2)**, Argentina **(B2)**, and New Zealand **(C2)**. Major reductions in SIDS mortality are not seen in NBW infants in the US (1995–2017), Argentina (1997–2015), and New Zealand (2000–2016).

To evaluate the progress of SIDS reduction over time, we plotted the rate of SIDS within the two distinct clusters based on birth weight from 1995 to 2017. Clinical guidelines, such as the Back to Sleep campaign, had a momentous effect on declining the SIDS rates in the US beginning in 1994, but the SIDS rate plateaued in the early 2000s (25). However, when calculating by birth weight, the rate of SIDS in infants <2,500 g has steadily decreased since 2008 (Figure 4A1—magenta). This may be due partly to the American Academy of Pediatrics' (AAP) safe sleep guideline describing the risks involved with bed-sharing. However, there is a significantly larger number of infants in the normal birth weight group (seen in Figure 4A2—green) that are not being equally impacted by implementing these clinical guidelines (Figure 4A1—green). Although the AAP's clinical guidelines are important, these discrete groups of SIDS decedents are not experiencing as rapid a reduction in incidence compared to the low birth weight group.

We broadened our analysis to see if the two distinct groups we identified had similar trends in other countries with different levels of cultural heterogeneity than the US (GDP per capita of $62,794.59) (42, 43). In both Argentina [lower cultural diversity compared to US with a GDP per capita of $11,683.95 (USD)] and New Zealand [similar cultural diversity to US and a GDP per capita of $41,945 (USD)], lower birth weight infants also have a higher rate of SIDS mortality compared to normal birth weight infants (Figures 4B1,C1—magenta). These data indicate that across three countries with different levels of cultural heterogeneity and GDP, similar trends in the rates of SIDS can be identified. In addition, the lower birth weight SIDS decedents are a much smaller number compared to normal birth weight SIDS decedents in all the countries (Figures 4B2,C2—green), and the rate at which this subgroup undergoes reduction in incidence rates over time has similarly been more rapid compared to the normal birth weight group. Note that New Zealand (Figure 4C) has been very successful in reducing mortality in the low birth weight group, with recent data from 2016, demonstrating complete overlap in the 95% confidence intervals of the fitted loess models of the normal birth weight and low birth weight data. Furthermore, the New Zealand low birth weight incidence has stabilized at ~0.6% mortality from 2012 to 2015, potentially indicating that the low birth weight trends may converge to the normal birth weight group. In summary, we conclude that major drivers in reduction of SIDS occurrences include reduction in mortality in low birth weight infants. These data suggest that additional insights into the developmental neuropathology of SIDS may be required to identify molecular pathways that could be targeted to prevent SIDS incidents.

## Discussion

### Autonomic Control and Postnatal Breathing Disorders

Autonomic control of breathing is impaired in human pediatric breathing disorders such as apnea of prematurity (AOP) (44, 45), Rapid Onset Hypoventilation Hypothalamic and Autonomic Dysfunction (ROHHAD) syndrome (46, 47), Rett syndrome (48), and Congenital Central Hypoventilation Syndrome (CCHS) (49). The full-term human brain doubles in size, reaching 75% of the adult brain weight by the end of the first postnatal year (50), and it is unclear how brainstem circuitry changes during this critical time period. Although anatomical deficiencies have been noted in the SIDS brainstem [such as arcuate nucleus hypoplasia (12)], it is unclear how other human brainstem structures function in homeostasis and if they are dysfunctional in SIDS. In this context, the study of known pediatric breathing disorders is particularly instructive. CCHS is a genetic pediatric breathing disorder caused by mutation in the gene *Phox2b*. Severe impairment in autonomic nervous system breathing control without cardiorespiratory disease is characteristic of CCHS (51, 52). Lack of CO_2_ chemosensitivity leads to diminutive tidal volumes and monotonous respiratory rates, resulting in rising blood pCO_2_ levels. Thus, the pathophysiology of CCHS may be instructive to understanding central pH/pCO_2_ chemosensation, and insights derived from CCHS are applicable to other pediatric breathing disorders. Human patients who die of CCHS in the perinatal period show neuronal loss of locus ceruleus as a common neuropathological finding (53, 54). Mouse studies have demonstrated that in severe forms of CCHS, developmental loss of visceral motor neurons result in non-cell autonomous deficiencies occurring in known brainstem respiratory pattern generators, including the CO_2_ chemosensitive retrotrapezoid nucleus and the preBötzinger complex, the main neonatal respiratory central pattern generator (55). Although such studies in rodents have been instructive in elucidating autonomic circuits, to date the study of rare genetic disorders such as CCHS and Rett syndrome has not resulted in knowledge that is generalizable to elucidating the molecular underpinnings of SIDS.

### Potential Neurodevelopmental Mechanisms Underlying SIDS

The intersection of neural, respiratory, and cardiovascular responses is at the forefront of SIDS mechanistic hypotheses. These hypotheses predict that the cause of death would be due to abnormal generation of respiration and preceded by multiple failures of protective mechanisms in the putative respiratory pathway (30, 56). These failures include a life-threatening event such as hypercapnia, hypoxia, asphyxiation in bedding, etc., and the failure of head lifting or turning. Some of these deficits are due to well-known paradoxical physiological responses in newborn mammals. For instance, in all newborn mammals, acute hypoxia results in a rapid rise in ventilation driven mainly from carotid bodies (Phase I) followed by a CNS-mediated repression of the ventilation (Phase II/Roll-off) which overshoots baseline and can even be elicited using brainstem slices (57). Recent studies in mice have shown that exposing newborn mouse pups to complete anoxia results in reduction in heart rate and respiration and does not recover correctly when *Pet1*-derived neurons, which generate a large population of 5HT neurons, undergo silencing (58). Along these lines, an infant could undergo failure of arousal, hypoxic coma, bradycardia and gasping, and failure of autoresuscitation (30). Emergency brainstem reflexes should trigger a rescue response to reinstate eupnea, however, certain disease states could impair this response, resulting in death (59). In premature infants, episodes of apnea are prevalent and separated into two categories: awake apnea and sleep apneas. SIDS decedents are often found following a period of sleep; however, it is still unknown whether death occurred during the sleep phase or following brief arousal.

The most common cause of sleep apneas in premature infants are respiratory pauses associated with the laryngeal chemoreflex (LCR). The LCR is characterized by a preceding period of hypoxia with bradycardia, swallowing, and a cough to clear the airway of obstructive fluids. Discovered in piglets, the swallowing and coughing response during the LCR exhibits low complexity values, signifying it is induced by a homogenous group of neurons (60). Piglets early in age also exhibited shorter duration of respiratory activities, suspected to be due to the rapid development of this neuronal system within the first month of life (60). In the event that a vulnerable infant has an apneic occurrence and they have susceptibility in the homogenous neuronal population controlling for the LCR, this puts the infant in a position they cannot survive (60).

In addition to the rapid development of the neurons controlling the LCR postnatally, the majority of postnatal proliferating cells in the pons are oligodendrocytes (61, 62). Prenatal insults might impact the differentiation of oligodendrocytes, leading to the failure of myelination of important pathways spanning the length of the brainstem. An extensively studied pathway in regards to SIDS is the serotonergic (5-HT) pathway. Effector nuclei responsible for cardiorespiratory integration, pharyngeal and laryngeal airway control, respiratory rhythm generation, and parasympathetic and sympathetic innervation may be impacted by the lack of myelination. In addition, whether the 5-HT abnormalities are causative and have pathological significance or correlative and a genetic predisposition in this pathway in a subset of SIDS decedents is yet to be determined (63).

### Recommendations for Forensic Neuropathology

In the US, sudden infant death cases fall under the jurisdiction of the local medicolegal investigation systems, which are currently a mixture of coroner, medical examiner, and hybrid systems. This heterogeneity of systems and investigative practices combined with funding limitations and a national shortage of forensic pathologists has led to inconsistencies in the certification of sudden, unexpected deaths in infants (64, 65). In addition, there has been increasing reluctance by forensic pathologists to certify a death as SIDS or SUID, partly due to some forensic pathologists viewing SIDS is a diagnosis of exclusion, making it difficult to diagnose if there are any limitations in the scene investigation, lack of clear circumstances around the death, or if external risk factors, such as unsafe sleep conditions, are present. As a result, there has been a shift toward using “Undetermined” as the cause of death in many of these cases (65, 66). Unfortunately, these variations in cause of death classification directly impact the ICD-10 codes used by epidemiologists and researchers, as SIDS is given an R95 code and “Undetermined” is given an R99 code.

In 2019, the National Association of Medical Examiners' (NAME) Panel on Sudden Unexpected Death in Pediatrics, an expert panel composed of medical examiners, pediatricians, and federal agency representatives, released “Unexpected Pediatric Deaths: Investigation, Certification, and Family Needs” which recognized this issue and the need for consensus guidelines for the investigation and certification of unexpected pediatric deaths (67). As such, they put forth recommendations for a standardized system of completing death certificates for these cases. Sudden, unexpected infant deaths that remain unexplained after a thorough investigation and autopsy were recommended to be certified as “Unexplained Sudden Death” as the cause of death (thereby generating an R95 ICD-10 code) with an additional indication of whether intrinsic factors (defined as natural conditions or risk factors associated with abnormal physiology) or extrinsic factors (defined as conditions in the infant's immediate environment that are a potential threat to life but cannot be deemed the cause of death with reasonable certainty) were present. The specific intrinsic and/or extrinsic factors are not entered on the death certificate, but rather the NAME Panel proposed they be incorporated into a synoptic report that would become a component of the autopsy report. Synoptic reports are commonly used in surgical pathology reports for cancer diagnoses and have allowed for increased consistency across pathologists and institutions, however synoptic reporting has not been widely used in the forensic setting. Recognizing the desire to capture the complex information regarding unexpected pediatric deaths, the NAME Panel's recommended synoptic report includes information regarding the extent of the investigation, medical history of the infant, specifics of the autopsy procedures performed, toxicology, ancillary studies, and radiologic studies. Thus, not only is the synoptic report a useful resource for capturing data but it also highlights any limitations of the investigation or autopsy so that these factors can be considered in data analyses. Furthermore, they had specific recommendations for when to use “Undetermined” as the cause of death. “Undetermined (Not otherwise specified)” should be used for deaths where the cause of death cannot be determined due to circumstances or findings that may raise uncertainty about the manner of death, those that were not considered to be sudden deaths, or those with competing causes of death. Cases where the cause of death could not be determined because of substantial limitations in the investigation or autopsy examination were recommended to be certified as “Undetermined (Insufficient Data).” While this classification system would still not capture all the potential SIDS cases (particularly in systems where resources and funding are limited for investigations and autopsies), it does provide more guidance to forensic pathologists that would hopefully allow for more consistency in reporting.

## Data Availability Statement

The data analyzed in this study is subject to the following licenses/restrictions: privacy or ethical restrictions imposed by the United States Centers for Disease Control. Requests to access these datasets should be directed to nvssrestricteddata@cdc.gov.

## Author Contributions

JB conceptualized and designed the study, carried out analysis, and drafted the initial manuscript. VC obtained Argentine infant mortality data, reviewed, and revised the manuscript. JS and JZ refined the analysis, reviewed, and revised the manuscript. AS and CP critically reviewed and revised the manuscript. JO conceptualized the study, refined analysis, reviewed, and revised the manuscript. All authors approve the final manuscript as submitted.

## Conflict of Interest

The authors declare that the research was conducted in the absence of any commercial or financial relationships that could be construed as a potential conflict of interest.

## Footnotes

^1^Centers for Disease Control and Prevention, Data and Statistics for SIDS and SUID.

^2^Centers for Disease Control and Prevention. National Center for Health Statistics, Public Use Data File Documentation:1990–2017 Period Linked Birth/Death Data Sets, Hyattsville, MD: Department of Health and Human Services, 1990–2017.
