# Supplementary material for: Revisiting the Neuropathology of Sudden Infant Death Syndrome (SIDS)
Source: Front Neurol. 2020 Dec 17;11:594550. doi: 10.3389/fneur.2020.594550 (PMC7773837; doi:10.3389/fneur.2020.594550)
Supplement: Supplementary file 1 [file Table_1.DOCX]

**Supplemental Table 1- Mother’s Education**

| **Mother’s Education Recoded for Analysis** | **Unrevised Birth Certificate** | **Revised Birth Certificate** |
| --- | --- | --- |
| 1= No formal education; Elementary school or less (≤ 8^th^ grade) | 00 = No formal education  01-08 = Elementary School | 1 = 8^th^ grade or less |
| 2 = Attended some high school but not graduated | 09-11 = 1-3 years of high school | 2 = High school grades 9^th^-12^th^ with no diploma |
| 3 = Assuming mother graduated after 4 years of high school (unrevised) or graduated/GED (revised) | 12 = 4 years of high school | 3 = High school graduate or GED completed |
| 4 = Some college, less than a bachelor’s degree | 13-15 = 1-3 years of college | 4 = Some college credit, not a degree  5 = Associate Degree (AA, AS) |
| 5 = Attended 4 years of college (unrevised) or attained a Bachelor’s Degree (revised) | 16 = 4 years of college | 6 = Bachelor’s degree (BA, AB, BS) |
| 6 = 5+ years of college (Master’s, Doctorate, Professional Degree) | 17 = 5+ years of college | 7= Master’s degree (MA, MS, MEng, MEd, MSW, MBA)  8 = Doctorate (PhD, EdD) or Professional Degree (MD, DDS, DVM, LLB, JD) |
| 99 = Unknown/Not stated | 99 = Unknown  Blank = Not on certificate | 9 = Unknown  Blank = Not on certificate |

**Supplemental Table 2- Variables Used for t-SNE**

| Infant birth information | Birth year, birth month, day of week- birth, gestation, birth weight, plurality |
| --- | --- |
| Mother’s information | Mother’s age, race/bridged race, education, marriage status at birth* |
| Prenatal care | Month prenatal care began, number of prenatal care visits, birth facility |
| Infant death information | Death year, month of death, number of health conditions at death, age at death (legal or recoded based on gestation) |

*imputed in 72 instances for 2017 data

**Supplemental Table 3- Linear Model of Race Related to GDP**

| **Race** | **Intercept** | **GDP Coefficients** | **GDP p** | **Adjusted R-Squared** |
| --- | --- | --- | --- | --- |
| White | 5.027e^-1^ | -2.056e^-7^ | 0.0322 | 0.07628 |
| Black | 7.635e^-1^ | -2.286e^-8^ | 0.922 | -0.02152 |
