# Supplementary material for: Revisiting the Neuropathology of Sudden Infant Death Syndrome (SIDS)
Source: Front Neurol. 2020 Dec 17;11:594550. doi: 10.3389/fneur.2020.594550 (PMC7773837; doi:10.3389/fneur.2020.594550)
Supplement: Supplementary file 2 [file Table_2.DOCX]

Supplemental Materials – Code for R

*To be posted to github upon acceptance*

#load csv data of each year

#determine variables consistently collected and recode so they are consistent over the time frame – In earlier years, yes is coded as “1”, in the revised birth certificate, yes is “Y”

Combine data.frames and recode variables as factors

Large_file<-rbind(small_data_1990, small_data_1991, small_data_1995, …, small_data_2017)

Large_file$VARIABLE<-as.factor(Large_file$VARIABLE)

Generate t-SNE model

Library(Rtsne)

Library(ggplot2)

Library(RColorBrewer)

Tsne_model_1 = Rtsne(data_tsne, check_duplicates = FALSE, pca = FALSE, perplexity = #, theta = 0.5, dims = 2, max_iter = 3000)

tsne<-plot(Tsne_model_1$X1, Tsne_model_1$x2, col = data_tsne$VARIABLE)

my_colors = c(brewer.pal(name = “#pick palette”, n = 8)

plot(tsne_model_df, aes(x = V1, y = V2, colour = data_tsne#VARIABLE)) +

scale_color_manual(values = my_colors)+

theme_bw()

#plot Violin boxplot based on 3 clusters

#generate column of age corrected for gestation based on formula in methods

#group cluster designation in column $Group

Library(ggplot2)

v<-ggplot(SIDS, aes(x = Group, y = age_corrected_for_gest, fill = Group))+

geom_violin()+

theme_bw()

v+coord_flip()+geom_boxplot(width = 0.1)+theme(axis.text.y = element_text(angle = 45))

#compile rates of SIDS occurrence by birthweight in each state

#center-scale data

Library(ggplot2)

Library(viridis)

Library(maps)

Library(mapproj)

Library(dplyr)

Library(ggthemes)

us_states<-map_data(“state”)

P<- ggplot(data = us_states, mapping = aes(x = long, y = lat, group = group))

P+geom_polygon(color = “gray90, size = 0.1)+

Coord_map(projection = “albers”, lat0 = 39, lat1 = 45)+

Guides(fill = FALSE)

states_rates$region <-states_rates$state

states_rates$region<-tolower(states_rates$state)

us_states_rates<-left_join(us_states, states_rates)

states_rates_lbw_scaled2<-join(us_states, states_rate_lbw_scaled)

p0<-ggplot(data = states_rates_lbw_scaled2, Year > 1994,

mapping = aes(x = long, y = lat, group = group, fill = Rate))

p1<-p0+geom_polygon(color = “gray90”, size = 0.05)+

coord_map(projection = “albers”, lat0 = 39, lat1 = 45)

p2<-p1+ scale_fill_viridis_c(option = “plasma”)

p2 + theme_map()+facet_wrap(~Year, ncol = 3)+

theme(legend.postition = “bottom”,

strip.background = element_blank())+

labs(fill = “SIDS Death Rate Scaled Within Birth Weight Category”)
