# Supplementary figures and images for: Revisiting the Neuropathology of Sudden Infant Death Syndrome (SIDS)
Source: Front Neurol. 2020 Dec 17;11:594550. doi: 10.3389/fneur.2020.594550 (PMC7773837; doi:10.3389/fneur.2020.594550)

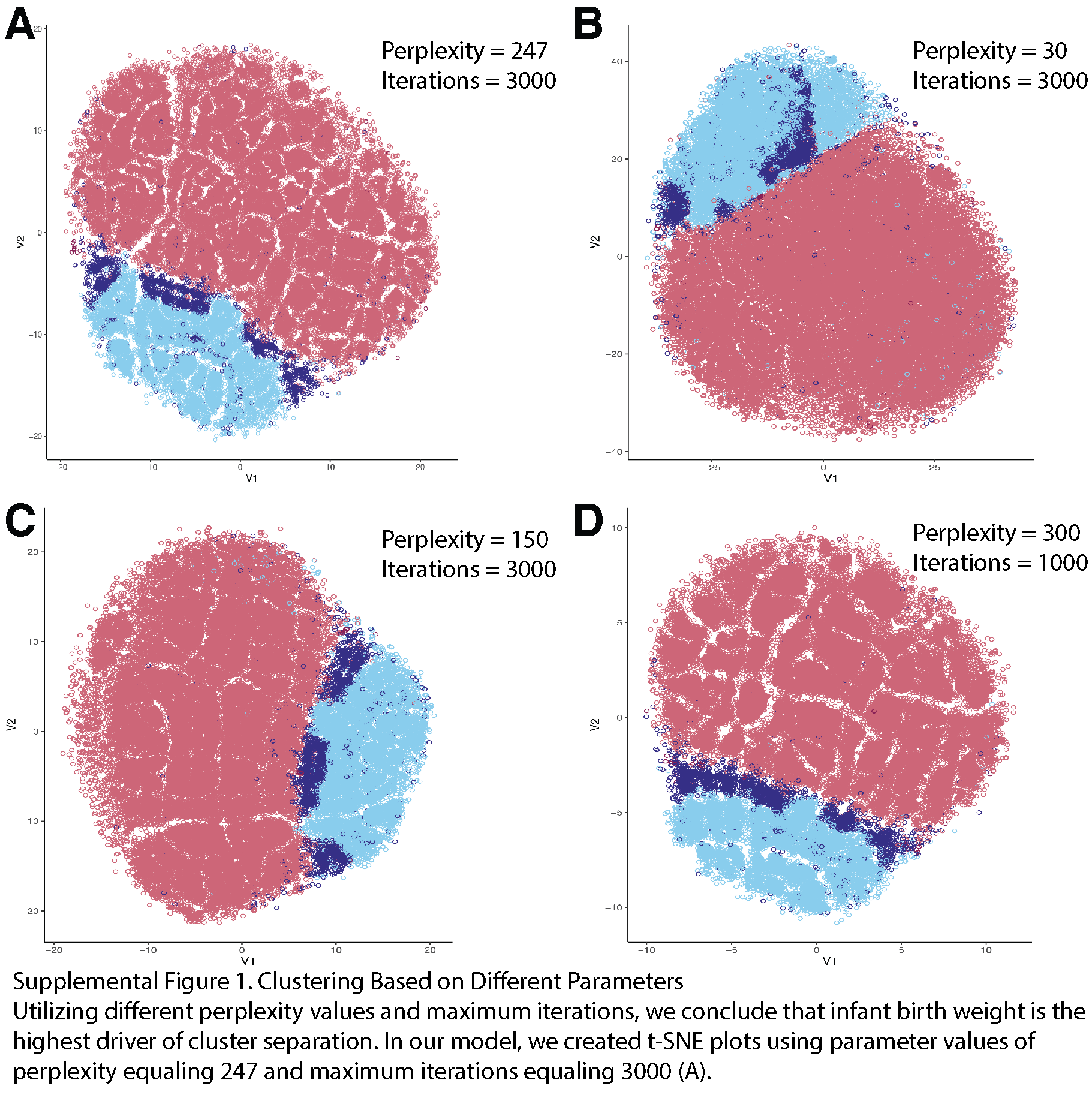

Supplement: Supplementary file 3 [file Image_1.TIF]

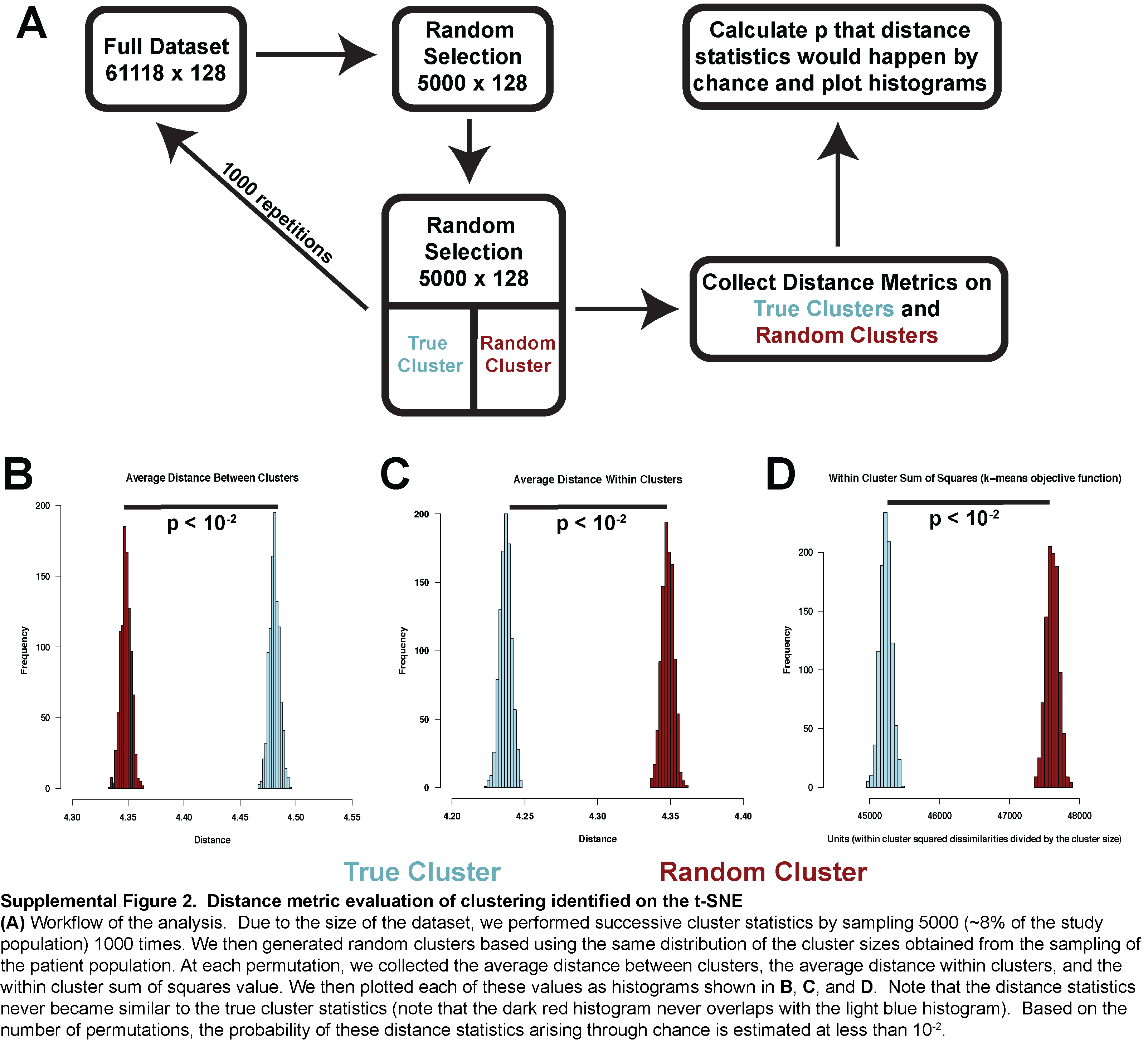

Supplement: Supplementary file 4 [file Image_2.TIF]
